# Which Is the Most Suitable Classification for Colorectal Cancer, Log Odds, the Number or the Ratio of Positive Lymph Nodes?

**DOI:** 10.1371/journal.pone.0028937

**Published:** 2011-12-13

**Authors:** Yong-Xi Song, Peng Gao, Zhen-Ning Wang, Lin-Lin Tong, Ying-Ying Xu, Zhe Sun, Cheng-Zhong Xing, Hui-Mian Xu

**Affiliations:** Department of Surgical Oncology and General Surgery, First Hospital of China Medical University, Shenyang, People's Republic of China; The Chinese University of Hong Kong, Hong Kong

## Abstract

**Objective:**

The aim of the current study was to investigate which is the most suitable classification for colorectal cancer, log odds of positive lymph nodes (LODDS) classification or the classifications based on the number of positive lymph nodes (pN) and positive lymph node ratio(LNR) in a Chinese single institutional population.

**Design:**

Clinicopathologic and prognostic data of 1297 patients with colorectal cancer were retrospectively studied. The log-rank statistics, Cox's proportional hazards model, the Nagelkerke R^2^ index and a Harrell's C statistic were used.

**Results:**

Univariate and three-step multivariate analyses identified that LNR was a significant prognostic factor and LNR classification was superior to both the pN and LODDS classifications. Moreover, the results of the Nagelkerke R^2^ index (0.130) and a Harrell's C statistic (0.707) of LNR showed that LNR and LODDS classifications were similar and LNR was a little better than the other two classifications. Furthermore, for patients in each LNR classification, prognosis was homologous between those in different pN or LODDS classifications. However, for patients in pN1a, pN1b, LODDS2 and LODDS3 classifications, significant differences in survival were observed among patients in different LNR classifications.

**Conclusions:**

For patients with colorectal cancer, the LNR classification is more suitable than pN and LODDS classifications for prognostic assessment in a Chinese single institutional population.

## Introduction

Colorectal cancer is the third most common cancer for both males and females, as well as the second leading cause of cancer-related death in the western world [1]. In China, with improvements in living standards and changes in diet, the incidence of colorectal cancer is gradually increasing [2]. Recently, the incidence of colorectal cancer and its cancer-related mortality have become the fourth highest of all cancers in China [3]. As is well known, lymph node (LN) metastasis is one of the most important prognostic factors in patients with colorectal cancers [4]–[10].

In the 7^th^ edition of the UICC/AJCC TNM staging system, based on the number of tumor-infiltrated lymph nodes, the pN category was stratified into pN_1_ (1–3 positive LNs) and pN_2_ (≥4 positive LNs) [4]. The lymph node ratio (LNR), namely, the ratio of positive LNs divided by the total number of retrieved LNs, reflects the probability of positive LNs in the retrieved LNs [5]. Recently, the LNR has been reported to represent a powerful independent prognostic value in colorectal cancer 5–10. Interestingly, another novel prognostic indicator, log odds of positive lymph nodes (LODDS), has been proposed in recent years. LODDS is defined as the log of the quotient of the number of positive lymph nodes and the number of negative lymph nodes and has been introduced as a new prognostic factor in breast cancer research [11], [12]. Moreover, Wang et al. studied 24,477 patients with stage III colon cancer who were registered in the Surveillance Epidemiology and End Results (SEER) database and revealed that LODDS was a better prognostic factor than LNR [13]. However, to date, no study comparing the prognostic value among pN, LNR and LODDS classifications for colorectal cancer in Chinese patients has been reported.

In light of these considerations, the aim of the current study was to investigate which is the most suitable classification among pN, LNR and LODDS classifications in prognostic assessment for colorectal cancer patients with R0 resection in a Chinese single institutional population.

## Materials and Methods

### Patients

From our prospective database, clinical information on all patients with colorectal cancer that underwent surgery at the Department of Surgical Oncology at the First Hospital of China Medical University from April 1994 to December 2007 were retrospectively collected, reviewed, and analyzed. No previous local or systemic treatment had been conducted for these patients before operation. Specimens which were fixed in formalin and stained with hematoxylin-eosin (H&E) were used for histopathological evaluation. This study consisted of stage I–III colorecal cancers. Patients (i) who died in the postoperative period (within 30 days), (ii) with multiple adenocarcinomas of the colon and rectum, (iii) with synchronous or metachronous tumors, (iv) who underwent neoadjuvant treatment due to presumed treatment-related changes in the TNM classification, (v) with incomplete pathological data entries, (vi) who were lost to follow-up, (vii) with tumor deposits, and (viii) distant metastasis were excluded in this study. Follow-up was completed for the entire study population until November 2008.

Of the remaining 1297 patients, the median and mean follow-up periods were 47 months and 56±36 months (range: 1–167 months), respectively. The following data were obtained: age, gender, date of surgery, date of death (if applicable), cause of death (if applicable), date of follow-up, location of the primary tumor, tumor size, histologic grade, venous invasion, lymphovascular invasion, depth of invasion, number of retrieved lymph nodes and number of metastatic lymph nodes. Tumors originating from the cecum to the sigmoid colon were defined as colon cancers and tumors located in the rectum or rectosigmoid junction were considered as rectal cancers [14].

### Ethics statement

The study was approved by the Research Ethics Committee of China Medical University, China. Written informed consents were obtained from all patients before participating in the study.

### Classification methods and Statistical Analysis

According to the 7^th^ edition of the UICC/AJCC TNM staging system, based on the number of tumor-infiltrated lymph nodes, the pN category was stratified into pN0: no positive LNs; pN1a: 1 positive LN; pN1b: 2–3 positive LNs; pN2a: 4–6 positive LNs; and pN2b: ≥7 positive LNs [4]. LNR was defined as the ratio of positive LNs divided by the total number of retrieved LNs, reflecting the probability of positive LNs in the retrieved LNs, which does not significantly depend on the number of LNs harvested [5]. LODDS was estimated by: log 
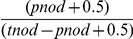
, where the pnod is the number of positive lymph nodes and tnod is the total number of lymph nodes retrieved, and 0.5 is added to both numerator and denomination to avoid singularity [13].

To obtain optimal cut-off values for LNRs and LODDS classifications, running log-rank statistics was applied [15]. Cancer-specific survival was analyzed by Kaplan-Meier survival curves and comparisons were made by the log-rank test. Multivariate analysis was performed using backward stepwise Cox's proportional hazards model [16]. Three-step multivariate analysis was performed to investigate which N staging system had more potential to predict patient outcomes. The p-spline (Fitting Spline Models) function is used to fit a general spline term within the Cox model [17]. The Nagelkerke R^2^ index (R^2^_N_) was used to score the different Cox models [18]. R^2^ represents the proportion of variation explained by covariates in regression models [18], [19]. R^2^_N_ divides R^2^ by its maximum attainable value to scale it to within the range 0–1. R^2^_N_ is close to 1 for a perfectly predictive model, and close to 0 for a model that does not discriminate between short and long survival times. After each regression, a Harrell's C statistic was run to test the predictive capacity and fit of the model, respectively. A model with perfect predictive capacity (sensitivity and specificity of 100%) would have a Harrell's C statistic of 1.00 and the highest Harrell's C statistic was chosen as the best model [20].

All the statistical analyses and graphics were performed with the SPSS 17.0 statistical package (SPSS, Chicago, IL), Splus 8.0 (Insightful Corporation, Seattle, WA, USA) and STATA MP ver.10 (StataCorp LP, College Station, TX) statistical software. For all analysis, P<0.05 was considered significant.

## Results

**Figure 1:**
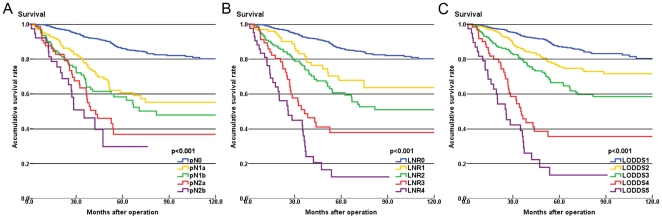
Survival curves of colorectal cancer patients according to three classifications (pN, LNR, LODDS) are depicted.

The number of lymph nodes examined in each specimen ranged from 1 to 107 with a mean of 13 and a median of 11. According to the 7^th^ edition of the UICC/AJCC TNM staging system, based on the number of tumor-infiltrated lymph nodes, the patients with different pN categories were divided into pN0: 935(72%); pN1a: 138(11%); pN1b: 121(9%); pN2a: 65(5%); and pN2b: 38(3%). The survival differences were statistically significant (P<0.001; Table 1 and Fig. 1A).

**Table 1:** Univariate analysis of the prognostic factors for patients with colorectal cancer.

	N^a^	5-YSR^b^(%)	P value*
Sex			**0.014**
Male	715	74	
Female	582	81	
Age			**0.003**
≤60	594	81	
>60	703	74	
Tumor location			0.931
Rectum	711	76	
Colon	586	78	
Tumor size			0.947
≤5	782	77	
>5	515	77	
Histologic grade			**<0.001**
Well	646	82	
Moderate	564	72	
Poor	87	63	
Venous invasion			0.701
Positive	7	67	
Negative	1290	77	
Lymphovascular invasion			**<0.001**
Positive	60	52	
Negative	1237	78	
pT stage			**<0.001**
T1	36	92	
T2	316	88	
T3	795	76	
T4	150	58	
pN stage			**<0.001**
N0	935	86	
N1a	138	61	
N1b	121	56	
N2a	65	37	
N2b	38	30	
LNR			**<0.001**
LNR0	935	86	
LNR1	99	68	
LNR2	164	59	
LNR3	57	38	
LNR4	42	12	
LODDS			**<0.001**
LODDS1	774	87	
LODDS2	223	75	
LODDS3	201	66	
LODDS4	61	36	
LODDS5	38	13	

N^a^: Number of patients.

5-YSR^b^: 5-year accumulative survival rate.

* : P values were made by log-rank test.

Using running log-rank statistics, we calculated the best cut-off LNR values and proposed a novel LNR category: LNR0: 0%; LNR1: 0%<LNR≤11%; LNR2: 11%<LNR≤36%; LNR3: 36%<LNR≤66% and LNR4>66%. Patients were categorized into five groups according to the LNR category: 935(72%) were as LNR0; 99(8%) were as LNR1; 164(13%) were as LNR2; 57(4%) were as LNR3 and 42(3%) were as LNR4. The 5-year cancer-specific survival rate decreased significantly with increasing LNRs: LNR0 = 86% survival rate; LNR1 = 68% survival rate; LNR2 = 59% survival rate; LNR3 = 38% survival rate; and LNR4 = 12% survival rate (P<0.001; Table 1 and Fig. 1B).

As shown in Table 1 and Fig. 1C, based on the LODDS classification, five groups were identified by running log-rank statistics: LODDS1≤−2.510; −2.510<LODDS2≤−1.680; −1.680<LODDS3≤−0.510; −0.510<LODDS4≤0.730; and LODDS5>0.730. The 5-year cancer-specific survival rates were 87%, 75%, 66%, 36% and 13%, respectively. The survival rate decreased significantly with increasing LODDS (P<0.001). Moreover, in univariate analysis, sex, age, histologic grade, lymphovascular invasion, and pT stage were also significantly correlated with prognosis (Table 1).

Then, we used univariate and three-step multivariate analysis (Cox Proportional Hazard Model) to find the most significant prognostic factors (Table 2). In univariate analysis, sex, age, histologic grade, lymphovascular invasion, pT stage, pN stage, LNR classification and LODDS classification were significant prognostic factors. Next, the step 1 multivariate analysis showed, pN classification, sex, age, histologic grade, lymphovascular invasion and pT classification were confirmed to be independent prognostic factors. After that, LNR classification was added to construct the model in the step 2 multivariate analysis, and LNR classification became significant, while pN classification and histologic grade dropped out of the model. Moreover, when all 3 N classifications were included in the step 3 multivariate analysis, LODDS and pN classifications were substituted by the LNR classification (Table 2).

**Table 2:** Univariate and Three-step Multivariate Analysis (Cox Proportional Hazard Model) of Prognostic Factors.

	*Univariate Analysis*			*Multivariate Analysis 1*			*Multivariate Analysis 2*			Multivariate Analysis 3		
	RR^a^	95% CI^b^	P	RR	95% CI	P	RR	95% CI	P	RR	95% CI	P
Sex, female vs male	0.730	0.567–0.940	0.015	0.714	0.555–0.920	0.009	0.762	0.592–0.982	0.035	0.762	0.592–0.982	0.035
Age*	1.016	1.005–1.027	0.004	1.023	1.012–1.034	<0.001	1.019	1.008–1.030	0.001	1.019	1.008–1.030	0.001
Tumor location, colon vs rectum	0.989	0.773–1.266	0.931									
Tumor size*	1.009	0.955–1.067	0.739									
Histologic grade, well vs moderate vs poor	1.521	1.264–1.829	<0.001	1.229	1.012–1.493	0.037						
Venous invasion, positive vs negative	1.312	0.326–5.277	0.702									
Lymphovascular invasion, positive vs negative	2.733	1.795–4.162	<0.001	2.193	1.409–3.414	0.001	2.603	1.691–4.008	<0.001	2.603	1.691–4.008	<0.001
pT stage, T1 vs T2 vs T3 vs T4	2.047	1.695–2.473	<0.001	1.764	1.446–2.152	<0.001	1.735	1.428–2.107	<0.001	1.735	1.428–2.107	<0.001
pN stage, N0 vs N1a vs N1b vs N2a vs N2b	1.796	1.646–1.961	<0.001	1.676	1.531–1.835	<0.001						
LNR, LNR0 vs LNR1 vs LNR2 vs LNR3 vs LNR4	1.915	1.759–2.086	<0.001				1.793	1.644–1.955	<0.001	1.793	1.644–1.955	<0.001
LODDS, LODDS1 vs LODDS2 vs LODDS3 vs LODDS4 vs LODDS5	1.939	1.765–2.130	<0.001									

RR^a^: relative risk.

CI^b^: confidence interval.

* : continuous variable.

**Figure 2:**
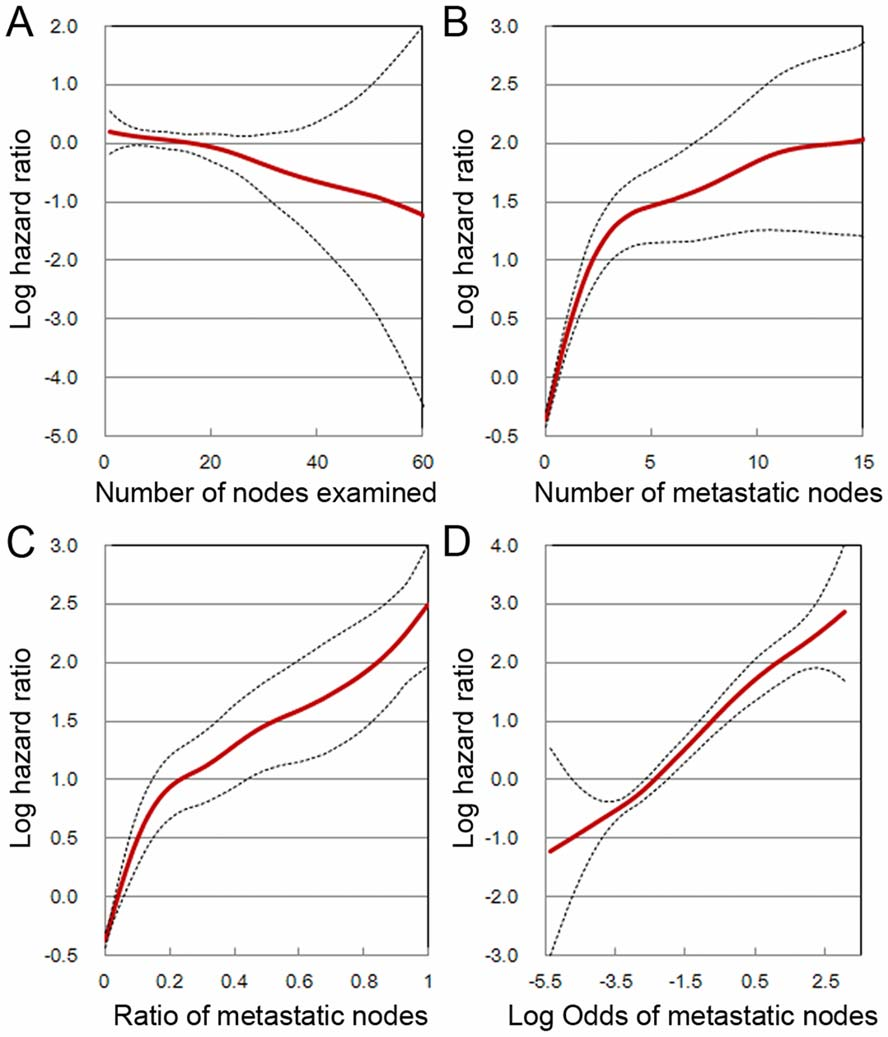
In fitting spline models, colorectal cancer mortality as a function of different classifications. Dotted lines indicate the 95% confidence interval.

Furthermore, in fitting spline models, the number of nodes examined and pN exhibited marked nonlinearity and widely diverging confidence intervals (Fig. 2A and 2B). The linearity improved for LNR and LODDS classifications, which also showed more homogeneously distributed confidence intervals (Fig. 2C and 2D).

Based on R^2^_N_, the results showed a comparison between proportional hazards models that included pN(R^2^_N_ = 0.100), LNR(R^2^_N_ = 0.130) and LODDS(R^2^_N_ = 0.119). The best predictive covariate model was LNR, obviously. Then, we used Harrell's C statistic to test the predictive capacity and fit of the model. The Harrell's C value and 95% CI of LNR (0.707, 0.675–0.739) and LODDS (0.708, 0.674–0.741) were similar and better than that of pN classification (0.698, 0.666–0.730). Comparing the predictive power of survival models with pN, LNR was significant (P = 0.002), but LODDS was not (P = 0.348). When we compared the predictive power between LNR and LODDS, there was no significant difference (P = 0.962).

Table 3 listed cancer-specific survival rates on the basis of pN and LODDS classification according to the LNR staging system. As shown, for patients in each LNR classification, prognosis was highly homologous between those in different pN or LODDS classifications. However, for patients in pN1a, pN1b, LODDS2 and LODDS3 classifications, significant differences in survival could always be observed among patients in different LNR classifications.

**Table 3:** Cancer-specific survival rates on the basis of pN and LODDS classification according to the LNR staging system.

	LNR0		LNR1		LNR2		LNR3		LNR4		P^a^
	N^d^	5-YSR^e^ (%)	N^d^	5-YSR^e^ (%)	N^d^	5-YSR^e^ (%)	N^d^	5-YSR^e^ (%)	N^d^	5-YSR^e^ (%)	
pN classification											
pN0	935	86	0		0		0		0		-
pN1a	0		84	66	46	61	5	20	3	0	<0.001
pN1b	0		15	82	83	61	13	57	10	0	<0.001
pN2a	0		0		28	53	26	29	11	25	0.150
pN2b	0		0		7	67	13	56	18	13	0.093
P^b^	-		0.409		0.972		0.719		0.115		
LODDS classification											
LODDS1	745	87	29	79							0.412
LODDS2	137	81	70	63	16	68					0.017
LODDS3	53	80			148	58					0.002
LODDS4							57	38	4	0	0.362
LODDS5									38	13	-
P^c^	0.346		0.153		0.471		-		0.884		

P^a^: Comparison of survival rates between different LNR groups.

P^b^: Comparison of survival rates between different pN groups.

P^c^: Comparison of survival rates between different LODDS groups.

N^d^: Number of patients.

5-YSR^e^: 5-year accumulative survival rate.

**Figure 3:**
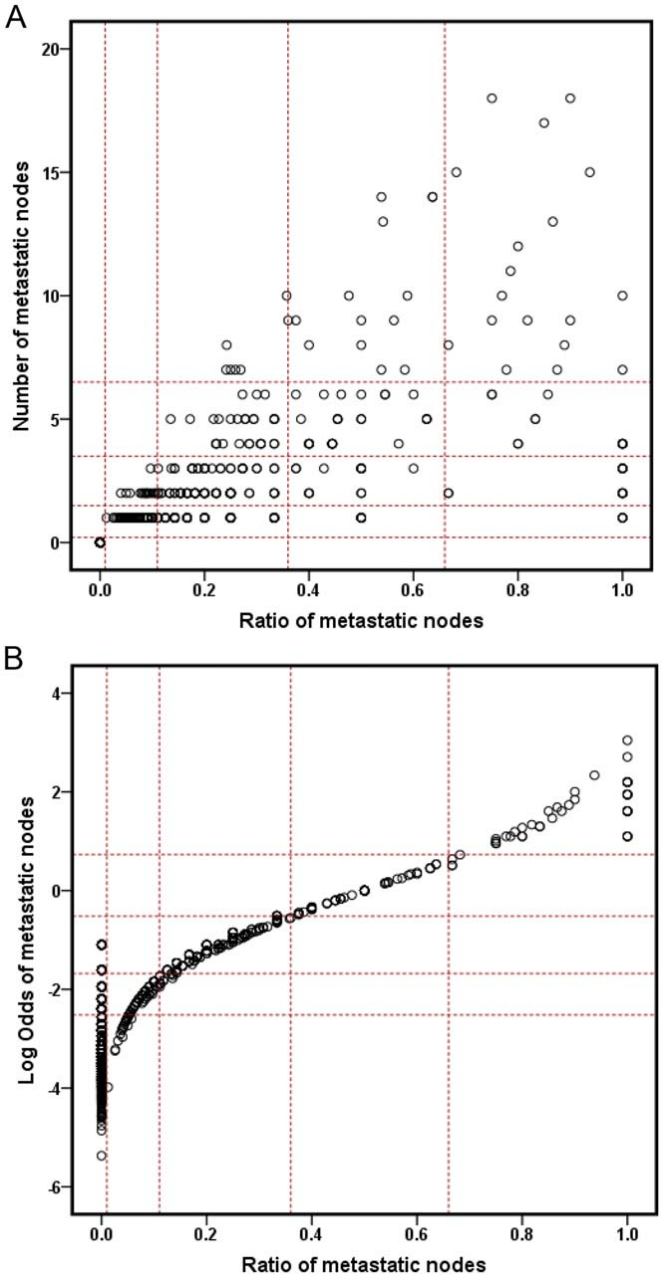
The distribution of the pN & LNR(A) and LODDS & LNR(B).

To explain why the LODDS classification was similar to LNR, we plotted scatter plots of the relationship among the three classifications. As shown in Fig. 3A, every pN classification can be divided into different LNR classifications. However, Fig. 3B showed that the patient distribution of LODDS classification was similar to the LNR classification and the value of LODDS increased with LNR increasing, indicating there was a close correlation between LODDS and LNR (except LNR = 0). When the LNR was 0, the value of LODDS was heterogeneous. However, Table 3 showed, for patients in the LNR0, prognosis was highly homologous between those in LODDS1, LODDS2 and LODDS3 classifications.

## Discussion

Although UICC/AJCC TNM classification was revised significantly from the 5^th^ edition to the 7^th^ edition, especially in regard to the pN categories [4], [21], [22], the pN categories still have some deficiencies. The primary flaw of the number-based UICC/AJCC pN classification is that the accuracy of the predicting prognosis was significantly influenced by the total number of nodes retrieved. According to the guidelines for colorectal cancer from the AJCC/UICC, only when the number of LNs that were retrieved and examined was 12 or more, it could be regarded as an adequate lymphadenectomy for accurate staging [4]. However, cases with insufficiently retrieved and examined LNs are not unusual in clinical practice. This led to the development and adoption of new prognostic indices that incorporate all the lymph node information in a single identifiable parameter. Among the indices, the important and promising classifications are the LNR and LODDS classifications [8], [13].

LNR has been identified as a significant prognostic value in breast cancer [23], pancreatic cancer [24], gastric cancer [25]. Furthermore, an increasing number of studies have demonstrated that the LNR classification is superior to the pN classification in colorectal cancer [5]–[10], [26]. LODDS, a novel indicator of predicting the status of lymph nodes, provides a new chance to improve the accuracy of N classification for prognostic assessment. But research on LODDS has mainly focused on breast and gastric cancer [11], [12], [27]. Only the study of Wang et al. revealed that LODDS was a better prognostic factor than LNR classification [13].

In our study, pN, LNR and LODDS classifications were all identified as significant prognostic factors in univariate analysis. To investigate whether one N classification was superior to the others, multistep multivariate analysis has often been used [27], [28]. For example, to prove the LNR classification was superior to the pN classification, we performed a three-step multivariate analysis. In the step 1 multivariate analysis, pN classification was one of the independent prognostic factors, whereas in the step 2 multivariate analysis, pN classification was substituted by the LNR classification. In addition, we performed a step 3 multivariate analysis, including all the 3 N classifications (pN, LNR and LODDS). The results indicated that the LNR classification was superior to both the pN classification and the LODDS classification. On the other hand, the results of the Nagelkerke R^2^ index and a Harrell's C statistic showed that LNR and LODDS classification were similar and LNR was a little better than the other two classifications.

LODDS classification divided the patients with negative lymph node into three groups: LODDS1, LODDS2 and LODDS3. In contrast,patients with negative lymph node were staged only as pN0 or LNR0 in pN or LNR classifications. Unfortunately, no significant survival difference was found among the patients in three LODDS classifications in the present study. Therefore, the prognostic effect of LODDS classification for negative lymph nodes colorectal cancer patients need further investigation in larger samples. Furthermore, our results further confirmed the superiority of the LNR classification: for patients in each LNR classification, prognosis was highly homologous among those in different pN or LODDS classifications. However, for patients in pN1a, pN1b, LODDS2 and LODDS3 classifications, significant differences in survival could always be observed among patients in different LNR classifications. Thus, we think the LNR classification is superior to the pN and LODDS classifications and it can contribute to accuracy in prognostic assessment.

To date, although a number of studies have shown that LNR classification was superior to the pN classification, no study comparing the prognostic value among pN, LNR and LODDS classifications for colorectal cancer in Chinese patients has been reported. In our study, we first demonstrated that the LNR classification was superior to the pN and LODDS classifications in 1297 Chinese patients with colorectal cancer. However, Wang et al. studied 24,477 patients with stage III colon cancer that were registered in the SEER database and revealed that LODDS was a better prognostic factor than LNR. It is possible that different cut off points acquired from different statistical methods for subclassification, different populations, different environments and different diet habits contribute to these different results.

In clinical practice, when the LNs that were retrieved and examined were insufficient, a so-called “stage migration” phenomenon [25] appeared due to inappropriate staging in the pN classification and the prognosis of the patient was underestimated. On the other hand, as the LNR classification is easier to calculate than the LODDS classification, LNR is recommended to be used in clinical practice.

Our study has some limitations. Our conclusion results from a Chinese single institutional study in 1297 patients with colorectal cancer. We used running log-rank statistics to calculate our cut-off values which were different from previous studies. Whether our results and cut-off values for LNR and LODDS can be applied to other institutions remains to be demonstrated. We look forward to performing larger sample studies and international multicentric research on LNR and LODDS classifications in colorectal cancer in the near future.

In conclusion, for patients with colorectal cancer, the LNR classification is more suitable than pN and LODDS classifications for prognostic assessment. Although the best and most clinically meaningful cut-off value for LNR classification has yet to be determined, we still believe that the LNR classification is the most reliable N classification to date and should be recognized in China in the future.
